# Inhibitory effect of catecholic colonic metabolites of rutin on fatty acid hydroperoxide and hemoglobin dependent lipid peroxidation in Caco-2 cells

**DOI:** 10.3164/jcbn.18-38

**Published:** 2018-07-12

**Authors:** Agustin Martin Morales, Rie Mukai, Kaeko Murota, Junji Terao

**Affiliations:** 1 Department of Food Science, Institute of Biomedical Sciences, Tokushima University Graduate School, 3-18-15 Kuramoto-cho, Tokushima 770-8503, Japan; 2 Department of Life Sciecne, Faculty of Science and Engeering, Kindai University, 3-4-1 Kowakae, Higashi-Osaka, Osaka 577-8502, Japan

**Keywords:** dietary rutin, lipid peroxidation, Caco-2 cell, catecholic colonic metabolite, colorectal cancer

## Abstract

To determine the preventive effect of dietary rutin on oxidative damages occurring in the digestive tract, 13-hydroperoxyoctadecadienoic acid and hemoglobin were exposed to Caco-2 intestinal cells after the pretreatment with colonic rutin metabolites. Among four catechol-type metabolites, quercetin and 3,4-dihydroxytoluene exerted significant protection on 13-hydroperoxyoctadecadienoic and hemoglobin-dependent lipid peroxidation of this epithelial cell. Compared with quercetin, a much lower concentration allowed 3,4-dihydroxytoluene to maximize the protective effect, though it needed a longer pre-incubation period. Neither quercetin nor 3,4-dihydroxytoluene affected the expression of peroxiredoxin-6 protein, which comprises the cellular antioxidant defense system. It is concluded that 3,4-dihydroxytoluene is a plausible rutin colonic metabolite that can suppress oxidative damages of intestinal epithelial cells by directly inhibiting lipid peroxidation. This result may illuminate the preventive role of dietary rutin against colorectal cancer incidence in relation to the consumption of red and processed meat.

## Introduction

Lipid peroxidation occurring ubiquitously in the tissues and biological fluids has been implicated in exerting both deleterious and beneficial effects on human health.^(1,2)^ In particular, non-enzymatic lipid peroxidation happens readily in the digestive tract because food-derived pro-oxidants such as peroxidized lipids and hemoproteins can meet to induce the generation of free radicals responsible for the initiation of lipid peroxidation in epithelial cell membranes.^(3,4)^ Interestingly, recent meta-analyses revealed the correlation between colorectal cancer incidence and consumption of red and processed meat.^(5,6)^ It has been suggested that hemoprotein-induced lipid peroxidation is involved in the etiology of colon carcinogenesis based on the consumption of red and processed meat.^(7,8)^ Angeli *et al.*^(9)^ indicated the combination of lipid hydroperoxides, primary lipid peroxidation products, and hemoglobin enhanced lipid peroxidation as well as DNA damage in a colon cancer cell line.

**Fig. 1:**
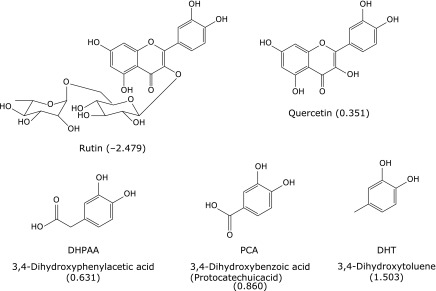
Structures and ClogP values of rutin and its colonic metabolites. Lipophilicity was calculated with ChemBio3D Ultra 14.0 software package (PerkinElmer, UK) on the basis of the chemical formula of the compounds and determined as a logarithm of the partition coefficient in n-octanol/water.

Rutin (3,3',4',5,7-pentahydroxyflavone-3-rutinoside), a glycosylated form of quercetin, is a plant flavonoid; its main sources are buckwheat, parsley, tomatoes, wine and apricots. In contrast to quercetin aglycone and its glucosides, rutin is hardly absorbed at the site of the small intestine.^(10,11)^ Dietary rutin mostly enters into the large intestine, where colonic microflora liberate quercetin from rutin, which can be absorbed or further degraded to produce various ring-scission products.^(12–14)^ Dietary flavonoids have long been suggested to exert a cancer-preventive effect by a variety of molecular mechanisms including antioxidant action, although the exact mechanism occurring *in vivo* is not yet known.^(15)^ It is therefore of much interest to evaluate the inhibitory effect of rutin colonic metabolites on lipid peroxidation occurring in the colonic epithelial cells from the viewpoint of prevention of colorectal cancer incidence. 3,4-Dihydroxyphenylacetic acid (DHPAA) and 3,4-dihydroxybenzoic acid (protocatecuic acid: PCA) are well-investigated as major human colonic metabolites of rutin.^(12–14)^ 3,4-Dihydroxytotulene (DHT) was also found in human urine and serum.^(16,17)^ Colonic microflora is implied to be involved in its production from rutin.^(18)^ Figure 1 shows the structures of these metabolites and their hydrophobicity calculated by a logarithm of the partition coefficient calculation (ClogP).^(19)^ These catechol-type metabolites are known to possess strong antioxidant activity by scavenging free radicals efficiently.^(20,21)^

The purpose of this study is to evaluate the antioxidant effect of these catechol-type colonic metabolites of rutin on lipid peroxidation occurring in the colon epithelial cells. 13-Hydroperxyoctadecdienoic acid (13-HpODE) and hemoglobin were used as a model of food-derived initiators of lipid peroxidation in the digestive tract. Differentiated Caco-2 cells, that is, human epithelial colorectal adenocarcinoma cells, were exposed to 13-HpODE and hemoglobin after the pretreatment of these metabolites. The effect of rutin colonic metabolites on intestinal peroxiredoxin-6 (Prx-6), a representative antioxidative enzyme,^(22)^ was also discussed.

## Materials and Methods

### Chemicals

13-HpODE was purchased from Cayman Chemical (Ann Arbor, MI). Rutin, quercetin, DHPAA, PCA, DHT and human hemoglobin were obtained from Sigma-Aldrich (St. Louis, MO).

### Cell culture

Caco-2 cells were obtained from the American Type Culture Collection. Caco-2 cells were grown in 75 cm^2^ flasks (Greiner Bio-one, Kremsmünster, Austria) in the presence of Dulbecco’s modified eagle medium (DMEM) containing 15% heat-inactivated fetal bovine serum (FBS) (Gibco Life Technologies, Gaithersburg, MD) and 1% nonessential amino acid (NEAA), penicillin (50 U/ml) and streptomycin (50 µg/ml). Seeding density was 1 × 10^4^ viable cells/cm^2^ with a 7 days passage frequency in a ratio 1:4 for subculture. Cells were incubated at 37°C in a humidified atmosphere of air/carbon dioxide (95:5, v/v) and the medium was changed (10 ml) every 48 h. For the experiment, cells were seeded at 90% confluency, and grown on 6-well, 12-well or 96-well plates (Thermo Scientific, Waltham, MA) in the presence of DMEM containing 15% heat-inactivated FBS, 1% NEAA, penicillin (50 U/ml) and streptomycin (50 µg/ml). Medium was changed every 48 h for 2 weeks to get a differentiated cell monolayer.

### Lipid peroxidation reaction of Caco-2 cells

Rutin and rutin metabolite solutions were prepared in methanol and further diluted in DMEM. Cells were pretreated with rutin metabolites (final conc. 30 µM) at 37°C for 24 h, and then washed with PBS. 13-HpODE and hemoglobin were diluted in DMEM. Then, cells were treated with 13-HpODE solution (final conc. 25 µM) and hemoglobin solution (final conc. 50 µM) separately and together and incubated at 37°C for another 24 h. As a negative control, methanol at equivalent volume (0.2% in the medium) was added to the blank sample.

### Lipid peroxidation assay in Caco-2 cells

 Thiobarbituric acid (TBA) reaction was carried out according to the method described by Fukunaga *et al.*^(23)^ with a few modifications. Briefly, cell samples in the 6-well plate were recovered in 100 µl of ice cold PBS and sonicated for 30 s. Samples (each 25 µl) were added to 100 µl of 0.2% TBA and incubated for 60 min in a boiling water bath. After samples were cooled down on ice, 100 µl of butanol were added to the mixture and centrifuged at 4°C for 5 min (18,000 *g*). Finally, samples were filtered through 0.45 µm filter and 20 µl of the sample was injected into HPLC column. The HPLC column was TSK-gel ODS-80Ts (4.6 mm × 150 mm). Eluting solvent was composed of methanol and water (50:50, v/v) and the flow rate was set at 0.7 ml/min. The eluate was monitored with fluorescence detection (ex. = 515 nm, em. = 553 nm). Tetraethoxypropane was used as a malondialdehyde equivalent and adopted for the standard curve of TBA reactive substances (TBARS). Protein content was determined by the methods of Bradford.^(24)^

### Western blot analysis

After the treatment, cells were washed twice with cold PBS and collected in 100 µl of lysis buffer [50 mM Tris·HCl pH 7.5, 150 mM NaCl, 5 mM EDTA, 1% Triton X-100, protease inhibitor (Complete EDTA-free; Roche, Basel, Switzerland), and phosphatase inhibitor (PhosSTOP; F. Hoffmann-La Roche, Basel, Switzerland)]. After 30 s sonication and centrifugation (4°C, 30 min), the protein amount of the supernatant was determined by bicinchoninic acid assay and samples were frozen at –30°C until western blot analysis.

Lysates were diluted in accordance with protein amount, mixed with 6× Laemmli buffer (Bio-Rad, Hercules, CA) and boiled at 100°C for 5 min. Samples were electrophoresed on polyacrylamide gels in the presence of sodium dodecyl sulfate (13% SDS-PAGE) and then electroblotted to immobilon transfer membranes (IPVH00010, Millipore, Bedford, MA). The membranes were washed 3 times in Tris-buffered saline containing 0.05% Tween-20 (TBST) and incubated for 1 h in commercial blocking buffer (Blocking-One; Nacalai Tesque, Kyoto, Japan), followed by overnight incubation at 4°C in primary antibodies: anti-Nrf2 (1:1,000 dilution; Santa Cruz Biotechnology, Dallas, TX), anti-peroxiredoxin 6 (1:500 dilution; Abcam Inc., Cambridge, MA) and β-actin (1:1,000 dilution; Cell Signaling Technology Inc., Danvers, MA). The membranes were then washed 3 times in TBST and incubated for 1 h with peroxidase-labeled secondary antibodies. Finally, the membranes were washed in TBST and protein bands were visualized with a chemiluminescence detection kit (ECL prime; GE Healthcare, Chicago, IL) using a LAS-3000 UV Mini Luminescent Image Analyzer (Fujifilm, Tokyo, Japan). NF-E2-related factor 2 (Nrf-2) and Prx-6 bands were quantified using ImageJ software (National Institutes of Health, Bethesda, MD) and normalized to that of β-actin.

### Data analysis

Reported values represent means ± SE (*n* = 3). Statistical analysis was evaluated by one-way or two way ANOVA followed by Tukey multiple comparison test to identify significantly different means (*p*<0.05).

## Results

### Effect of pretreatment of rutin metabolites on 13-HpODE and hemoglobin dependent lipid peroxidation of Caco-2 cells

**Fig. 2:**
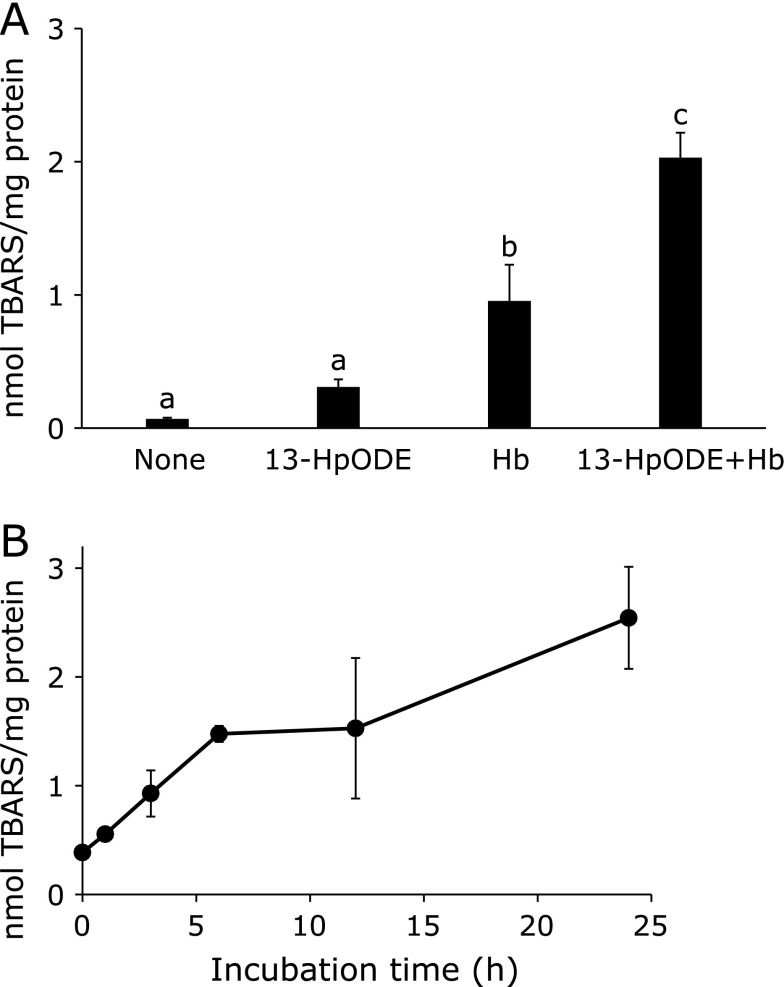
Lipid peroxidation of Caco-2 cells induced by 13-HpODE and/or hemoglobin. (A) Effect of 13-HpODE and/or hemoglobin (Hb) on lipid peroxidation level after incubation at 37°C for 24 h. (B) Time course of lipid peroxidation up to 24 h in the presence of both 13-HpODE and hemoglobin. In each experiment, cells were treated with 13-HpODE (25 µM) and/or hemoglobin (50 µM). The contents of TBARS were expressed as nmol malondialdehyde/mg protein. Values are the mean ± SE from independent triplicate determinations for each experiment. Different letters represent significant differences, which were evaluated by two-way ANOVA with the Tukey multiple comparison test (*p*<0.05).

**Fig. 3:**
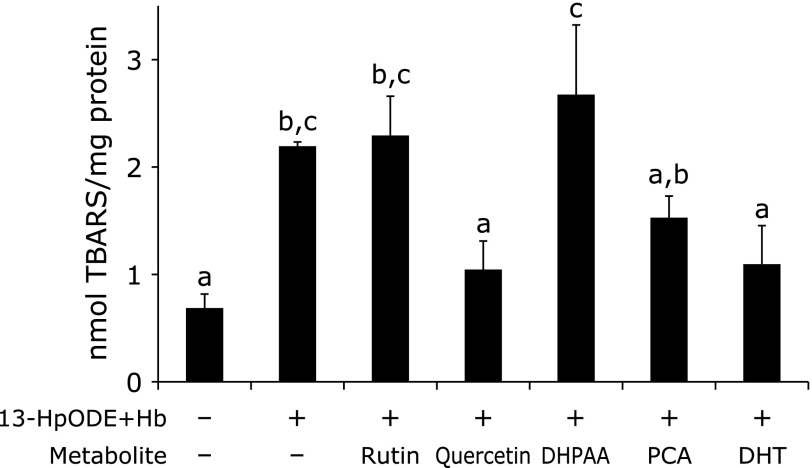
Effect of rutin and its colonic metabolites on13-HpODE and hemoglobin-induced lipid peroxidation of Caco-2 cells. Cells were pre-treated with test compounds (30 µM) for 24 h at 37°C. After addition of 13-HpODE (25 µM) and hemoglobin (Hb: 50 µM), cells were further incubated at 37°C for 24 h. The contents of TBARS were expressed as nmol malondialdehyde/mg protein. Values are the mean ± SE from independent triplicate determinations for each experiment. Different letters represent significant differences which were evaluated by two-way ANOVA with the Tukey multiple comparison test (*p*<0.05).

Figure 2A shows the lipid peroxidation level of Caco-2 cells measured by the content of TBARS after incubation with 13-HpODE (25 µM) and/or hemoglobin (50 µM) for 24 h. The combination of 13-HpODE and hemoglobin obviously enhanced lipid peroxidation of this cultured cell. Lipid peroxidation level was increased time-dependently up to 24 h (Fig. 2B). At 24 h incubation, cell viability was found to be kept more than 80% and then remarkably decreased to 70% after 48 h incubation (data not shown here). Thus, the incubation period of 24 h was adopted to measure the effect of rutin metabolites on 13-HpODE and hemoglobin dependent lipid peroxidation of Caco-2 cells. Pre-incubation of quercetin and DHT at 30 µM for 24 h produced a significant protective effect against lipid peroxidation, whereas none of the other metabolites exerted any effect under the same experimental conditions (Fig. 3).

### Comparison of the effect of quercetin and DHT on the inhibition of 13-HpODE and hemoglobin dependent lipid peroxidation of Caco-2 cells

**Fig. 4:**
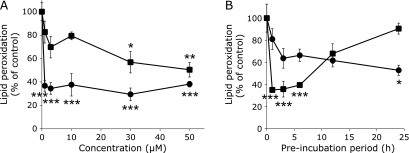
Comparison of the inhibitory effect on lipid peroxidation of Caco-2 cell between quercetin and DHT. (A) Concentration-dependent inhibition by quercetin and DHT. ■, quercetin; ●, DHT. Cells were pretreated with quercetin or DHT at different concentration for 24 h. (B) Pretreatment time-dependent inhibition by quercetin and DHT. Cell were pretreated with quercetin (30 µM) or DHT (30 µM) for different incubation periods. In each experiment, 13-HpODE solution (final conc. 25 µM) and hemoglobin solution (Hb: final conc. 50 µM) were added to the cell medium after pretreatment time and then incubated for 24 h. 13-HpODE and hemoglobin solutions were freshly prepared just before addition to each cell medium. Values are the mean ± SE from independent triplicate determinations for each experiment. Significant differences from initial values before incubation are indicated by *p<0.05, **p<0.01 and ***p<0.001 with one-way ANOVA and Tukey multiple comparison test.

The effect of pre-incubation with quercetin and/or DHT on 13-HpODE and hemoglobin dependent lipid peroxidation of Caco-2 cells was compared at different concentrations in the range between 1 and 50 µM. Significant inhibition appeared at more than 30 µM for quercetin and more than 1.0 µM for DHT after 24 h incubation (Fig. 4A). Figure 4B shows the effect of pre-incubation period on the inhibition of lipid peroxidation by quercetin and DHT at 30 µM. Quercetin was highly effective in inhibition at incubation period for 1–6 h. Interestingly, the inhibitory effect decreased after 12 h of incubation and for up to 24 h after. In contrast, DHT exhibited an inhibitory effect in a time-dependent manner for up to 24 h. These results indicate that the maximum inhibition by quercetin and DHT were obtained at a different concentration and pre-incubation period. Compared with quercetin, DHT needs a longer pre-incubation period but a much lower concentration for maximizing the inhibitory effect on 13-HpODE and hemoglobin dependent lipid peroxidation.

### Effects of quercetin and DHT on Nrf-2 and Prx-6 protein levels

**Fig. 5:**
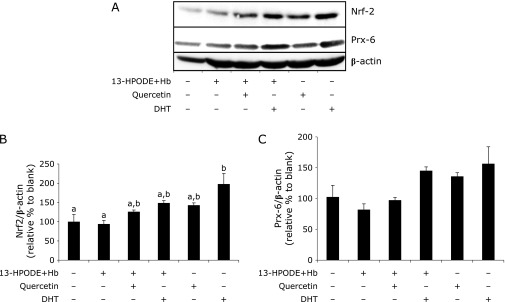
Effect of quercetin and DHT on the protein levels of Nrf-2 and Prx-6 in Caco-2 cells in the presence or absence of 13-HpODE/hemoglobin. (A) Western blot analysis and (B) quantification of the band density. Caco-2 cells were pretreated with quercetin (30 µM) or DHT (30 µM) for 24 h at 37°C. Then, cells were incubated for another 24 h with or without 13-HpODE (25 µM) and hemoglobin (Hb: 50 µM) at 37°C. Levels of Nrf-2 and Prx-6 proteins were quantified using western blotting analyses as prescribed in the Materials and Methods section. Density was quantified and normalized to that of β-actin. Values are the mean ± SE from independent triplicate determinations for each experiment. Different letters represent significant differences, which were evaluated by two-way ANOVA with the Tukey multiple comparison test (*p*<0.05).

Figure 5 shows the result of western blot analysis of Nrf-2 and Prx-6 expressed in Caco-2 cells after the pretreatment of quercetin or DHT and subsequent incubation with or without 13-HpODE and hemoglobin. Incubation of the cells with 13-HpODE and hemoglobin *per se* did not affect these protein levels. DHT significantly elevated the Nrf-2 protein level when cells were treated without 13-HpODE and hemoglobin. However, no significant difference was found in the effect of these two compounds on Prx-6 protein level irrespective of the treatment of 13-HpODE and hemoglobin.

## Discussion

Here we found that the combination of 13-HpODE and hemoglobin enhanced lipid peroxidation in the Caco-2 cell line. It is likely that heme-Fe(III) and heme-Fe(IV) react with the hydroperoxy group of 13-HpODE to produce lipid peroxyl radicals and lipid alkoxyl radicals responsible for initiating lipid peroxidation in the epithelial cells.^(9)^

Maestre *et al.*^(25)^ demonstrated that oxidized products of polyunsaturated fatty acids (PUFA) can be accessible to Caco-2 cells, though the uptake of the oxidation products was ~10% of the uptake of unoxidized PUFA. Muller *et al.*^(26)^ found that 13-HpODE was reduced to hydroxyl derivatives in the Caco-2 cell monolayer by glutathione peroxidase activity if applied to its luminal side. Therefore, lipid peroxidation-initiating radicals seem to be generated at the cell surface, where 13-HpODE reacts with heme-Fe(III)/Fe(IV) of hemoglobin, leading to the promotion of autocatalytic lipid peroxidation in cellular membranes.

The results concerning the effect of rutin, quercetin, DHPAA, PCA and DHT on 13-HpODE and hemoglobin dependent lipid peroxidation of Caco-2 cells (Fig. 3) demonstrated that only quercetin and DHT were effective in the suppression of lipid peroxidation of the intestinal epithelial cells. We previously found that lipophilic quercetin, but not hydrophilic quercetin glucosides, can be easily absorbed into Caco-2 cells and suggest that the lipophilicity plays a crucial role in the absorption of quercetin derivatives into intestinal epithelial cells.^(27)^ Thus, hydrophilic property of rutin may interrupt their absorption, leading to reduced antioxidant function in the epithelial cells. PCA and DHPAA also exerted little effect in the suppression of lipid peroxidation probably because of their ionized carboxylic group, which disturbs their incorporation into the cells.

Figure 4 shows that DHT exerts a stronger protective effect on lipid peroxidation at a much lower concentration than quercetin, although it needs a longer incubation period at 30 µM. In contrast, quercetin was more effective than DHT at this high concentration with a shorter pre-incubation period (1~6 h). This phenomenon may be derived from the low stability of quercetin in the cells in spite of its high cellular uptake efficiency. In fact, we previously found that quercetin disappeared completely from cell fraction after incubation with model nerve cells for 24 h.^(28)^ DHT seems to be a more stable compound than quercetin. It is therefore likely that DHT is an active metabolite of rutin in the intestinal tract.

Su *et al.*^(29)^ found that DHT effectively inhibited inducible nitric oxide synthase and cyclooxygenase-2 in lipopolysaccharide stimulated murine macrophages. Furthermore, several studies have proposed a variety of biological functions of DHT as rutin colonic metabolites.^(30–33)^ Interestingly, Furukawa *et al.*^(34)^ reported that DHT was able to up-regulate the expression of heme oxygenase-1 in neural stem/progenitor cells. Thus, DHT may exert modulating effect on redox sensitive transcriptional factors of antioxidative enzymes, resulting in the enhancement of antioxidant defense in the epithelial cell line, together with a direct free radical-scavenging effect. Therefore, we selected Prx-6, a lipid hydroperoxide-reducing enzyme, as a target of Caco-2 cell antioxidant difference, because Prx-6 was suggested to be involved in the antioxidant defense of murine intestinal mucosa.^(35)^ Here we found that Prx-6 and its transcription factor, Nrf-2,^(36)^ were certainly expressed in these epithelial model cells. However, we could not find the enhancement of the Prx-6 protein level by exposure to DHT irrespective of the presence of 13-HpODE/hemoglobin. Thus, it is not clear whether this intestinal metabolite is capable of modulating Prx-6 expression in the intestinal epithelial cells.

In conclusion, among catechol-type colonic metabolites of rutin, DHT was identified as a possible candidate to suppress oxidative damage of intestinal epithelial cells. Further research is necessary to clarify concentration and behavior of DHT in the colon after rutin intake from the viewpoint of preventing red meat and processed meat-related colorectal cancer incidence.

## Abbreviations

DHPAA: 3,4-dihydroxyphenylacetic acid
DHT: 3,4-dihydroxytoluene (4-methylcatechol)
DMEM: Dulbecco’s modified eagle medium
FBS: fetal bovine serum
13-HpODE: (±)13-hydroperoxy-9*Z*,11*E*-octadecadienoic acid
Nrf-2: NF-E2-related factor 2
PCA: protocatechuic acid
Prx-6: peroxiredoxin 6
PUFA: polyunsaturated fatty acid
TBA: 2-thiobarbituric acid
TBARS: thiobarbituric acid reactive substances
TBST: Tris-buffered saline-containing 0.05% Tween-20
